# Self-care model and body image in adults after a bariatric surgery

**DOI:** 10.1590/1518-8345.5592.3536

**Published:** 2022-07-08

**Authors:** Gisela Pineda-García, Aracely Serrano-Medina, José Manuel Cornejo-Bravo, Víctor Hugo Andrade-Soto, Efraín Armenta-Rojas, Daniela Lilian González-Sánchez

**Affiliations:** 1 Universidad Autónoma de Baja Califonia, Facultad de Medicina y Psicología, Tijuana, Baja California, México.; 2 Universidad Autónoma de Baja Califonia, Facultad de Ciencias Químicas e Ingeniería, Tijuana, Baja California, México.; 3 Universidad Autónoma de Baja Califonia, Facultad de Ciencias de la Salud, Tijuana, Baja California, México.; 4 Bolsista do Consejo Nacional de Ciencia y Tecnología, México.; 5 Bolsista do Sindicato de Profesores Superación Universitaria de la Universidad Autónoma de Baja California, México.

**Keywords:** Self-Care, Body Image, Self-Efficacy, Obsession-Compulsion, Depression, Nursing, Auto-Cuidado, Imagem Corporal, Auto-Eficácia, Compulsão Obsessiva, Depressão, Enfermagem, Autocuidado, Imagen Corporal, Autoeficacia, Obsesión-Compulsión, Depresión, Enfermería

## Abstract

**Objective:**

the aim of the present article was to test a self-care model explained by the relationship between self-efficacy, body image, obsessive-compulsive disorder, and depression in people with bariatric surgery in the city of Tijuana, Baja California, Mexico.

**Method:**

this was a correlational cross-sectional study carried out between August and December 2020. Validated instruments were administered to 102 participants to measure their self-care capacity, general self-efficacy, psychopathological symptoms, and body image perception and satisfaction. The variables of interest were analyzed using descriptive statistics and the Pearson and Spearman correlation coefficients were used to develop a model using path analysis.

**Result:**

a significant model was obtained with adequate goodness-of-fit indicators (chi-square χ^2^ (8) = 11.451, *p* = .177; root mean square error of approximation (RMSEA)= 0.000; goodness-of-fit index (GFI)= 0.965; comparative fit index (CFI)= 0.985; parsimonious normed fit index (PNFI)= 0.509, and Akaike information criterion (AIC)= 37.451). Self-efficacy (Zβ=0.294) and body image dissatisfaction (So= -0.376) were shown to influence self-care abilities while psychopathological symptoms influenced body dissatisfaction: obsessive-compulsive disorder (Zβ=0.370) and depression (Zβ=0.320).

**Conclusion:**

adequate levels of self-efficacy and body satisfaction predict a high capacity for self-care.

## Resumo

**Objetivo:**

testar um modelo de autocuidado explicado pela relação entre autoeficácia, imagem corporal, transtorno obsessivo-compulsivo e depressão em pessoas com cirurgia bariátrica na cidade de Tijuana, no México.

**Método:**

estudo transversal correlacional realizado entre agosto e dezembro de 2020. Instrumentos validados foram aplicados a 102 participantes para medir sua capacidade de autocuidado, autoeficácia geral, sintomas psicopatológicos, e percepção e satisfação com a imagem corporal. As variáveis de interesse foram analisadas utilizando estatísticas descritivas e correlações de Pearson e Spearman para finalmente desenvolver um modelo utilizando a análise de caminho (*path analysis*).

**Resultados:**

foi obtido um modelo significativo com bons indicadores de bondade de ajuste (qui-quadrado χ 2 (8)= 11,451, p = 0,177; raiz quadrada da média do erro de aproximação (RMSEA)= 0,000; índice de bondade de ajuste (GFI)= 0,965; índice de ajuste comparativo (CFI)= 0,985; índice de ajuste normalizado parcimonioso (PNFI)= 0,509 e critério de informação de Akaike (AIC)= 37,451). A autoeficácia (Zβ= 0,294) e a insatisfação com a imagem corporal (Zβ= -0,376) demonstraram ter um efeito sobre as capacidades de autocuidado, enquanto os sintomas psicopatológicos influenciam a insatisfação corporal: transtorno obsessivo-compulsivo (Zβ= 0,370) e depressão (Zβ= 0,320).

**Conclusão:**

níveis adequados de autoeficácia e satisfação corporal predizem uma alta capacidade de autocuidado.

## Resumen

**Objetivo:**

probar un modelo de autocuidado explicado por la relación entre autoeficacia, imagen corporal, obsesión-compulsión y depresión en personas con cirugía bariátrica de la ciudad de Tijuana, B.C.

**Método:**

estudio transversal correlacional realizado entre agosto y diciembre de 2020. Se aplicaron instrumentos validados a 102 participantes para medir su capacidad de autocuidado, autoeficacia general, síntomas psicopatológicos, percepción y satisfacción de la imagen corporal. Las variables de interés fueron analizadas mediante estadística descriptiva y correlaciones de Pearson y Spearman para finalmente elaborar un modelo mediante análisis de ruta (*Path Analysis*).

**Resultados:**

se obtuvo un modelo significativo con buenos indicadores de bondad de ajuste (chi-cuadrado χ ^2^ (8)= 11.451, *p* = .177; error de aproximación raíz-media-cuadrada (RMSEA)= 0.000; índice de bondad de ajuste (GFI)= 0.965; índice de ajuste comparativo (CFI)= 0.985; índice de ajuste normado parsimonioso (PNFI)= 0.509 y criterio de información de Akaike (AIC)= 37.451). Se demostró que la autoeficacia (Zβ= 0.294) y la insatisfacción de la imagen corporal (Zβ= -0.376) tienen un efecto sobre las capacidades de autocuidado mientras que los síntomas psicopatológicos influyen sobre la insatisfacción corporal: obsesión-compulsión (Zβ= 0.370) y depresión (Zβ= 0.320).

**Conclusión:**

los niveles adecuados de autoeficacia y satisfacción corporal predicen una alta capacidad de autocuidado.

**Highlights**

(1) A high percentage of bariatric patients experience body dissatisfaction.

(2) All the patients sampled presented some degree of obsessive-compulsive disorder.

(3) Obsessive-compulsive disorder and depression were predictors of body dissatisfaction.

(4) Self-efficacy and body dissatisfaction explained self-care in bariatric patients.

## Introduction

In recent years, the increase in morbid obesity has gone hand in hand with the growth in the number of bariatric procedures, which have become the main treatment of this disease, with more and more people wanting to undergo this type of intervention^1^. According to the International Federation for Surgery for Obesity and Metabolic Disorders (IFSO), the United States performs the highest number of procedures (335,124 in 2019)^2^. 

About 28,000 people undergo bariatric surgery (BS) annually in Latin America, with rapid growth in the number of cases. This is attributed to the high obesity rates in the region^3^, coupled with the fact that Latin Americans who live in the United States and have obesity^4^ sometimes decide to return to their countries of origin to undergo this type of treatment, since Latin America has been a leader in bariatric surgery of comparable quality at a lower cost^5^. 

The success of bariatric surgery is explained because it allows for sustained long-term weight loss, achieving a decrease in morbidity and mortality and risk factors, in addition to optimizing anthropometric parameters and improving quality of life^6^. However, accelerated weight loss requires patients to be able to cope with and adapt quickly to their new body image (BI), with no time to reconstruct their own body representation. This leads to changes in how they relate to their environment. It has been observed that, in the early stages of the postoperative period, patients experience problems with the organization of their BI^7^.

Body image is a mental representation that each individual constructs in terms of feelings, attitudes and behaviors concerning their own body. It is a multi-dimensional construct that includes different components, such as perception (body size and silhouette), subjectivity (cognitive aspects and affective connection with the evaluation of one’s own body, resulting in satisfaction/dissatisfaction) and behavior (exposure, avoidance), where people with obesity have greater body image dissatisfaction (BID) than those without obesity^8^.

Body image dissatisfaction is a consequence of the discrepancy that results from the importance put on physical appearance by setting high beauty standards and evaluating one’s appearance as less attractive^9^. Bariatric patients often report an improvement in their BI; however, this is not the case for everyone, because for some, BI perception does not improve after surgery, with individuals expressing dissatisfaction with the aesthetic results of BS related to excess skin^10^ ^-^ ^11^. 

This dissatisfaction has a negative effect on emotions, generating distress or psychological discomfort such as depression because of the constant desire to achieve an unrealistic ideal figure that can even turn into obsessive-compulsive disorder (OCD)^12^. These emotions and thoughts can be damaging to levels of general self-efficacy and self-care ability, influencing patients to make decisions that put their health at risk, such as lack of adherence to prescribed treatment, which includes a balanced diet, moderate physical activity, consumption of bariatric multivitamin supplements, and follow-up visits^13^ ^-^ ^14^.

According to Bandura’s social cognitive theory, perceived self-efficacy is defined as “people’s judgments of their capabilities to organize and execute courses of action required to attain designated types of performance.” Bandura postulated that “a high sense of self-efficacy facilitates information processing and cognitive performance in diverse contexts, including decision-making”^15^. 

Self-efficacy levels are of great importance because they can increase or decrease motivation, which has been observed in health behaviors such as chronic disease management, exercise, weight loss, and in the ability to recover from health problems or avoid potential risks^16^. In these cases, patients with high perceived self-efficacy have a greater capacity for self-care^17^.

Self-care is the conceptual axis of Orem’s theory^18^, who defined it as “the set of actions that mature (or maturing) individuals carry out in the interest of staying alive and healthy, and continuing with personal development and well-being”. According to Orem, those who provide their own care have specialized skills, which were named “self-care capabilities”, which allow individuals to acquire new behaviors when they identify lifestyle imbalances that do not lead to an optimal level of self-care.

According to this theory, individuals exercise their self-care capabilities (“self-care agency”) by taking responsibility for their own care to maintain and improve a state of well-being and quality of life. Therefore, the “self-care agent” (patient) has the power to commit to a course of action and to perform activities to meet the ongoing requirements of self-care, which is known as “treatment self-management”.

According to the above, a possible limitation of “treatment self-management”^19^ in bariatric patients is the lack of preparation and acceptance of their new BI. Previous studies have shown a correlation between positive BI and adequate self-care capacity^20^ or a high level of self-efficacy^21^. According to the literature review, there is no scientific evidence that has studied these three constructs together in the bariatric population, which is of utmost importance because, as described, bariatric patients can feel unprepared for extreme psychosocial and lifestyle changes after surgery^22^.

Therefore, the present study aimed to test a self-care model based on the relationship between self-efficacy, BID, OCD, and depression among adults who underwent bariatric surgery in the city of Tijuana, Baja California, Mexico, with age and sex as covariates. The results of this study can help the development of follow-up treatment for bariatric patients.

## Method

### Study design

This was a cross-sectional correlational study.

### Sampling location

A private bariatric center in the city of Tijuana, B.C., Mexico.

### Period

This study was conducted between August and December 2020.

### Population 

The sample included individuals who had undergone bariatric surgery.

### Selection criteria

People of both sexes from 18 to 65 years old, with more than six months of having been operated with gastric sleeve techniques or Roux-en-Y gastric bypass, residents of the city of Tijuana, Mexico. People outside the age range, with other bariatric techniques and who had less than six months of surgery were excluded.

### Sample definition

The sample size was non-probabilistic, extracted from a database of n= 250 patients from a private bariatric medical center. After careful revision, those who met the inclusion criteria were contacted by phone by their surgeon, who was not part of the research team, to be invited to participate in the study. N= 180 patients met the inclusion criteria, of which n=22 could not be located and n=56 did not agree to participate. Therefore, the final sample consisted of n=102 people.

### Variables 

Exogenous variables were sex, age, surgical technique, date of surgery, self-efficacy and distress or psychological discomfort represented by depression and OCD. Body dissatisfaction and self-care capacity were included as endogenous variables.

### Data collection instruments

To collect sociodemographic and clinical data, a personal identification card was designed, whereas validated instruments were used to collect the variables of interest. The degree of distress or psychological discomfort was measured using the Symptom Checklist-90-Revised (SCL-90-R), created in 1977^23^ and modified in 1994. 

In 2005, it was translated into Spanish and validated for the Mexican population^24^, with a Cronbach’s alpha of >0.7-0.85. The Spearman range correlation values showed that, except for one item, all obtained a higher correlation value with their corresponding dimension: for 72%, the correlation was high (r>0.5) and for 26%, it was moderate (r>0.25 and <0.5). 

The scale consists of 90 items with Likert-type answer options ranging from 0 to 4 (0 = never; 1 = rarely; 2 = sometimes; 3 = frequently; 4 = always), where the patient responds to each item depending on the discomfort they have experienced in the week before the questionnaire, including the day of its administration. The scores for each factor are obtained by calculating the mean scores (sum of items divided by the number of items). 

The scale consists of nine subscales: somatization, obsessive-compulsive disorder, interpersonal sensitivity, depression, anxiety, hostility, phobic anxiety, paranoid ideation, and psychoticism. The total scale presented a Cronbach’s alpha of 0.98 and 70% of the variance explained in the sample data. For the purposes of this study, the obsessive-compulsive disorder (α= 0.89) and depression (α=0.86) subscales were reported. The cut-off scores were 1.5 for depression^25^, while OCD was categorized into three levels: very low (0 to 0.99), low (0.99 to 1.99) and high (>2.00).

Self-care capacity was measured using the Appraisal of Self-Care Agency Scale (ASA), which was translated into Spanish and adapted to be administered to the Latin population and used with people with obesity. It consists of 24 items with Likert-type answer options that include four options: completely disagree = 1, disagree = 2, agree = 3, and completely agree = 4. Scores range from 24 to 96. To assess the level of self-care capacity in the participating population, three categories were used: low capacity (<69 points), medium capacity (69 to 75 points) and high capacity (>76 points)^26^ ^-^ ^27^. The scale presented a Cronbach’s alpha of 0.82 and 42% of variance explained in the sample data.

General self-efficacy was measured using the Self-Efficacy Test, which measures a person’s perception about their ability to handle different stressful situations in their daily life.^28^. It has been validated in Spanish by various authors^29^ ^-^ ^30^ with a Cronbach’s alpha of 0.84, and an average intraclass correlation of 0.36. 

The questionnaire consists of 10 items with Likert-type answers that indicate how a person perceives each item in terms of their ability at the time of the test: false (1 point); barely true (2 points); somewhat true (3 points) or true (4 points), with a minimum score of 10 and a maximum of 40 points. The higher the score, the greater perceived general self-efficacy, with scores divided into two categories: low and high, with 28 points as the cut-off point^31^. The instrument showed good internal consistency in this sample (α 0.864, p<0.001) and according to previous studies, it has good validity, explaining 65.85% of the accumulated variance^32^. 

Body image was assessed using two measurement instruments, the Body Shape Questionnaire (BSQ) and the Body Image Assessment for Obesity (BIA-O), respectively. The BSQ was developed as a measure of preoccupations with body size and shape^33^ and consists of 34 items with Likert answer options: never=1, rarely=2, sometimes=3, often=4, very often=5 and always=6. The score ranges from 34 to 204, and body dissatisfaction is defined as a score greater than 110^34^.

The reliability and validity of this instrument have been tested and demonstrated in the Mexican population by various authors^35^ ^-^ ^36^, with a Cronbach’s alpha of 0.95 and an average correlation of *r*=0.395. In the sample data, this instrument obtained an internal consistency of 0.96 and 63% of explained variance. 

The BIA-O scale^37^ helps determine an individual’s degree of body dissatisfaction using 18 silhouettes numbered 1 to 18, ranging from very thin to very obese. Body satisfaction is calculated by subtracting the currently perceived figure from the ideal figure selected, where a test result >0 indicates the desire to be thinner, <0 corresponds to the desire to be larger and 0 indicates body satisfaction. This scale has recently been used in the bariatric population with good results^38^ ^-^ ^39^.

### Data collection

The patients were invited to participate voluntarily in the project and gave their written consent. The researchers collected their sociodemographic information and administered the instruments to measure the variables of interest in a single one-hour session.

### Data processing and analysis

Data processing and analysis were performed using the Statistical Package for the Social Sciences (SPSS), version 26 for Windows, which produced basic statistics for the description of the study and sociodemographic variables, as well as Pearson and Spearman correlations. In addition, exploratory factor analysis and Cronbach’s alphas were performed to determine the validity and reliability of the scales. Path analysis was carried out to obtain and test the model, using the AMOS 24 module.

The maximum likelihood estimation method was used to establish the model parameters. Goodness-of-fit was analyzed using chi-square (χ^2^), root mean square error of approximation (RMSEA), goodness-of-fit index (GFI), comparative fit index (CFI), parsimonious normed fit index (PNFI) and Akaike information criterion (AIC). 

Acceptable fit values for chi-square, GFI, CFI and PNFI are close to 1.0, acceptable RMSEA values are close to or less than 0.05, while lower AIC values indicate a better fit^40^ ^-^ ^42^. The results section presents the adjusted final solution, which includes the variables age, depression, OCD, BID measured with BSQ, self-efficacy and self-care. 

### Ethical aspects

This study was approved by the Bioethics Committee of the School of Medicine and Psychology of the Autonomous University of Baja California (Approval Number 1135/20-2), and carried out according to the Declaration of Helsinki of 1964^43^, its subsequent amendments, and in adherence to national and international ethical standards.

## Results

The sociodemographic data of the sample indicated that 90.2% of the participants were women, with an average age of M= 39.77, SD= 10.05, and 9.8% were men, with an average age of M= 40.9, SD= 5.64. 84.3% had undergone the gastric sleeve technique while the rest were subjected to Roux-en-Y gastric bypass. Regarding postoperative time, 50% had been operated one to three years prior to the study, followed by 27.5%, who had had the surgery six months to one year prior to the study, and 22.5%, with over three years, ranging between 6 months and 2 days to 16 years and 23 days.

Descriptive results are presented in Table 1, indicating that 78.5% of the participants reported adequate levels of self-care (61.8% with high capacity and 16.7%, with medium capacity) while 21.6% presented low capacity. The average was found in the high-capacity category (M=78.37, SD=8.25). Regarding self-efficacy, 83% presented adequate levels and 17% presented low self-efficacy. The average for this variable was above the cut-off point (M=33.7, SD=7.0). 

In relation to depression, only 12% fell above the cut-off point, indicating the presence of symptoms. For the OCD variable, most (73%) of the participants presented very low levels, 20% low levels, and only 7% presented high scores. The average score for OCD was in the very low category (M=.72, SD =.62).

**Figure 1:**
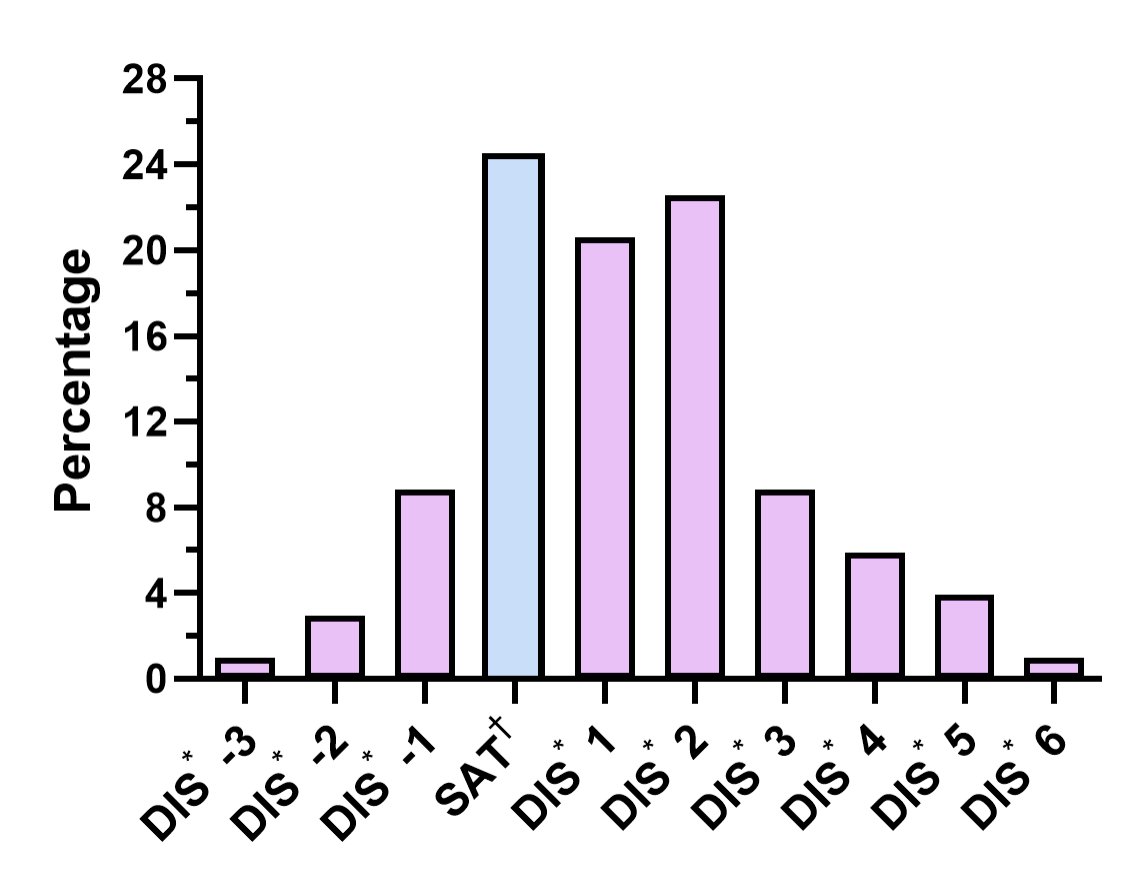
Body image dissatisfaction (BIA-O), total sample (n=102). Tijuana, B.C., Mexico, 2020

^*^DIS = Body image dissatisfaction; ^†^SAT = Satisfaction

As mentioned in the methodology, BI was measured using two scales. Regarding the BIA-O (Figure 1), the body image dissatisfaction (BID) scores indicated that a high percentage of the participants presented dissatisfaction and the desire to be thinner (62%). A lower percentage presented body image satisfaction (25%) and the desire to be larger (13%). According to the BSQ cut-off point, 44% of the respondents expressed a high level of negative attitudes towards their BI (Table 1).

**Table 1:** Descriptive statistics and prevalence of the variables BSQ, self-care, self-efficacy, depression, and OCD of the total sample (n^*^ = 102). Tijuana, B.C., Mexico, 2020

Variable	Category			Mean ± SD^†^
	Minimum		Maximum	
BSQ^‡^	55.9%		44.1%	101.58 ± 0.63
	<110		>110	
	Low capacity	Medium capacity	High capacity	
Self-care	21.6%	16.7%	61.8%	78.37 ± 8.25
	<69;	70-75	>76	
	Minimum		Maximum	
Self-efficacy	16.7%		83.3%	33.72 ± 7.0
	<28		>28	
	Absence		Presence	
Depression	88.2%		11.8%	0.69 ± 0.65
	<1,5		>1,5	
	Very low	Low	High	
OCD^§^	73%	20%	7%	0.72 ± 0.62
	0-0,99	1-1,99	>2	

^*^n = Number of cases; ^†^SD = Standard deviation; ^‡^BSQ = Body Shape Questionnaire; ^§^OCD = Obsessive-compulsive disorder

According to the results of the correlations between the sociodemographic variables and the measuring instruments presented in Table 2, a significant correlation was found between sex and BI measured by the BIA-O, which means that women presented better BI than men. On the other hand, BI measured by the BSQ was correlated with age, date of surgery, OCD, depression, and self-care. Therefore, advanced age, longer time elapsed since surgery, and good self-care capacity were related to greater body satisfaction, while high OCD and depression scores meant a higher BID.

**Table 2:** Correlation between sociodemographic variables and scores on measurement instruments (n^*^ = 102). Tijuana, B.C., Mexico, 2020

	*Age*	*Date of Surgery*	*Surgical Technique*	*OCD* ^*†*^	*Depression*	*Self-efficacy*	*Self-care*	*BSQ* ^*‡*^	*BIA-O* ^*§*^
**Sex**	-.032	.069	.142	-.090	.038	.012	-.095	-.025	.203^||^
**Age**	-	.371^||^	.265^||^	-.261^||^	-.279^||^	.064	-.058	-.302^||^	-.079
**Date of Surgery**		-	.180	-.141	-.134	.078	-.060	-.268^||^	-.066
**Surgical Technique**			-	.044	.051	-.132	.091	.041	.156
**OCD^†^**				-	.862^||^	-.110	-.266^||^	.646^||^	-.048
**Depression**					-	-.139	-.265^||^	.639^||^	.033
**Self-efficacy**						-	.352^||^	-.170	-.154
**Self-care**							-	-.419^||^	.015
**BSQ^‡^**								-	-.035
**BIA-O^§^**									-

^*^n = Number of cases; ^†^OCD = Obsessive-compulsive disorder; ^‡^BSQ = Body Shape Questionnaire; ^§^BIA-O = Body Image Assessment for Obesity; ^||^p<0.05

**Figure 2:**
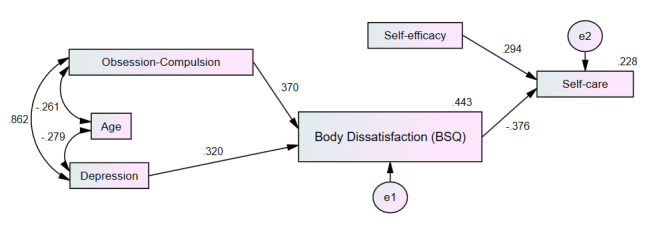
Self-care explained by self-efficacy and body dissatisfaction

Based on the results, a parsimonious model with adequate goodness-of-fit was developed (Figure 2), including the variables OCD, depression, age, BID measured by the BSQ, self-efficacy, and self-care. In this model, depression and OCD explain BID, and BID together with self-efficacy explain self-care. In the graph, the rectangles represent the observed variables, and the ovals represent the errors associated with the endogenous variables. The unidirectional arrows correspond to effects while the bidirectional ones display correlations. The goodness-of-fit measurements of the model are shown in Table 3.

**Table 3:** Goodness-of-fit measurements of the model (n^*^ = 102). Tijuana, B.C., Mexico, 2020

Absolute fit indices		Incremental fit index		Absolute fit indices	
Χ^2*^	^†^ RMSEA	^‡^ GFI	^§^ CFI	^||^ PNFI	^¶^ AIC
Χ^2*^=11.451 gl=8 p=0.177	0.000	0.965	0.985	0.509	37.451

^*^Χ^2^ = Chi-squared; ^†^RMSEA = Root mean square error of approximation; ^‡^GFI = Goodness of fit index; ^§^CFI = Comparative adjustment index; ^||^PNFI = Parsimonious normed fit index; ^¶^AIC = Akaike Information Criterion

The model shows negative correlations between the covariate age and depression (r=-0.279), and age and OCD (r=-0.279), indicating lower values with increasing age, as well as a high positive correlation between OCD and depression (r = 0.862, p <0.001), i.e., the greater the OCD, the greater the depression. Standardized regression weights indicated that for each higher increment of OCD and depression, negative BI (BSQ) increased by 0.370 and 0.320. respectively (p <0.05). The same figure shows that for each increased increment in self-efficacy and negative BI attitudes, self-care capacity increased by 0.294 and decreased by 0.376, respectively (p <0.001). Finally, the explained variance of BID was 44.3%, and that of self-care, 22.8%.

Initially, sex was considered as a covariate, however, the results indicated that it did not influence or correlate significantly with any variable in the model. This can be attributed to the fact that 90% of the sample was made up of women, so the effect of this variable could not be clearly established.

## Discussion

The sample was represented mainly by women, which can be attributed to the beauty standards that promote thinness, through the continuous exposure of models with this aesthetic standard. The impact of this “culture of thinness” can produce BID in people, especially women, since they tend to care more about their BI and therefore undergo weight-loss surgery more often to try to “fit” into social beauty standards^44^. In turn, this could explain the high prevalence of BID as measured by the BSQ in the sample and the desire of most of the sample to be thinner, as indicated by the BIA-O.

The final model obtained explained the participants’ self-care capacity based on BID, self-efficacy, OCD, and depression, in which age was a covariate. This is consistent with what was expected, since BID has a negative influence on self-care, while self-efficacy has a positive effect. However, because this model explained about 23% of the self-care variance, it is necessary to explore the influence of other variables on the self-care capacity in this population. 

In the proposed model, both self-efficacy and BI were essential to explain self-care in this population. It is important to study the self-care capacity in people who undergo BS, because the success of surgery largely depends on it. It is important to emphasize that people not only require skills, decision-making capacities, and commitment to face the new lifestyle and adaptation to a new BI, they also need to carry out treatment self-management to maintain a healthy weight^45^ and adhere to the prescribed treatment^46^, as this allows them to maintain well-being in health and avoid long-term complications.

Body image satisfaction has been associated with well-being, which is why there are proposals to include it in interventions to attempt to improve thoughts and feelings about weight through strategies that promote self-care^47^. In the present study, BI was a direct and negative predictor that explained self-care in the sample of patients who underwent BS: increased negative attitudes towards BI resulted in decreased self-care.

A possible strategy to promote self-care in these patients is to identify individuals who present negative psychosocial changes, especially those caused by BID. This should be addressed by trained health professionals, highlighting the importance of quality of life and mental well-being over physical appearance through preoperative guidance and continuous follow-up^48^. 

Some studies have evaluated the BI of people after having undergone a bariatric intervention^49^ and have found negative attitudes associated, among other variables, with symptoms of depression and lack of functionality due to excess “redundant” skin. The present study found that depression (in a significant association with the characteristics of OCD) explains the presence of greater negative BI attitudes among the participants.

In this sense, the high figures of dissatisfaction with BI found in the sample participants are striking. Only a small proportion wished to larger (people who perceived themselves to be too thin) and a larger percentage of people wished to be thinner, which was probably due to dissatisfaction with the excess of “redundant” skin, and lack of BI reorganization and redefinition demanded by their new physical appearance^50^ and the desire for a muscular slim silhouette among men and defined slim silhouette with salient breasts and buttocks among Mexican women^51^.

The influence of OCD characteristics on negative BI has been observed in young people^52^, and the same behavior of variables was observed in the sample of adult participants in the present study. In people with body dysmorphia, obsessive thoughts are associated with body appearance (such as the idea of having a larger body and face size); while compulsions consist of behaviors such as mirror avoidance, constantly checking one’s appearance, and frequently touching up makeup^53^. Among the general population, problems with BI have been related to low self-esteem, obsessive thoughts about appearance, and depression^54^. 

The classical scientific literature has identified a neurological relationship between OCD disorders and depression^55^, and similarly, this study corroborates these findings with a strong significant association between symptoms of OCD and depression in people who have undergone bariatric surgery. These disorders tend to occur more frequently in women who have undergone bariatric surgery^56^, so the percentage of participants with high scores on these scales can be explained by the sex distribution of the sample. Additionally, although the results of the measuring instruments do not constitute a clinical diagnosis of depression and/or OCD, they do help identify the presence of symptoms associated with these diseases because of the cut-off points established by previous studies. 

The results obtained in the present study suggest that similar further studies should be conducted to allow for the generalization of results in this population, because some of the main limitations of this research were the under-representation of men and heterogeneous distribution of surgical techniques, as these can influence the brain’s reward system, and therefore the BI self-evaluation and satisfaction^57^. Furthermore, it is advisable to evaluate the effect of surgery on body image satisfaction.

## Conclusion

The present study produced relevant results, because knowledge of the interaction between psychopathological symptoms (OCD and depression), BI and self-efficacy in the explanation of self-care among the bariatric population can help guide the development of interventions focused on promoting physical and mental health through positive changes in patient behavior, with the aim of improving BI perception, self-esteem, and self-care capacity.

Finally, the data reported in this study point to a strong association between symptoms of OCD and depression that function as predictors of negative BI attitudes. Self-efficacy in collaboration with BI explains self-care in people who have undergone bariatric surgery.
